# Foul Breath

**Published:** 1879-04

**Authors:** J. T. Codman


AMERICAN JOURNAL

OF

DENTAL SCIENCE.

Vol. XII. THIRD SERIES—APRIL, 1879. No. 12.

### ARTICLE I.

### *Foul Breath.*

BY J. T. CODMAN, D. M. D.

(An Essay read before the joint meeting of the Mass. Dental Society and the American Academy of Dental Science. Boston, Jan., 1879. Published by vote of the latter Society.)

The subject of which I propose to treat to-day is not an inviting one. It is shunned by most writers, and articles treating of it in dental journals are very rare. The question naturally arises in our minds, what is the reason of this almost total neglect of this very important topic, which, if not one absolutely belonging to our specialty, is one so intimately connected with it that it should interest every member of it.

I cannot but think that the neglect is occasioned by want of that knowledge of its primary causes, and a lack of general knowledge of the relation of all the organs, one to another, that work together for the sustenance and maintainance of the life and health of our corporate frames; and that this want pervades both the dental and medical professions.

For what other reason could such an important subject be ignored? Does it not intrude itself upon us everywhere—in the street, in the coach or car in which we travel, and in our homes? Are we not annoyed at every turn—at the place of amusement, at the church and the lecture? Ofttimes we meet the intelligent and the able, the profound and the artistic. They may have earnest and noble faces; their conversation be rapt and energetic; but we turn away in disgust from the foul odor that comes in the same breath with words of affection, of wisdom and love. Are not our hearts pained within us, and is not our pity even greater than our disgust for the unfortunates who are thus afflicted? Must they not know that we turn away from them, and that they are shunned for no other reason than this of impure breath?

The love of purity and sweetness is inherent in our natures. It is impossible for us to remain in the closest intimate relation of personal friendship or love with those who belch forth foulness. There must, in such cases, be personal and mental reservation. A sweet breath, then, becomes a fortune and a blessing, and woe to the pretty face! woe to the loving heart! woe to the striving one who longs for higher position, for more of love or more of caress! who has it not.

But, as a class, whom doth it annoy more than the dentist, who, hour after hour and day after day, earns his bread by bending over the "gateway of life"—the breathing places of human beings—taking to his central vitality the putrid odors that emanate from the unsounded depths of foulness? But, alas for the weakness of humanity! the truth, and the whole truth (for it must be told) is, we who criticize are not exempt from this plague. Verily, have I not been offended beyond measure, in dental meetings and among dentists, by dentists themselves? Therefore should we attend to this subject; therefore should we wake from our sleepiness and supineness for our own good, and, curing ourselves, be ready to go forth to cure all others.

It being a subject connected with the primary functions of life and health, it is necessary to know something of primary functions, to understand the mode of cure; for it is a disease which unfortunately afflicts all occasionally, from the infant to the oldest person; and the paucity of knowledge of the philosophy of physiology must be the reason of the neglect of its cure.

Doubtless the causes of bad breath are numerous. Temporarily, it may be produced by any nauseous or ill-flavored thing, either chewed or used as food, as garlics, onions, snakeroot, tobacco, etc., or equally by bad-odored drinks, by simple apposition to the teeth and mouth, by diseases of the teeth—common caries—with the odor of decaying tooth substance and the remnants of food left within the cavities getting foul, also by diseased gums, which emit a peculiarly unpleasant smell.

All these troubles are embraced within one cause, which cause itself is a reason for foul breath; namely, want of cleanly habits; and all yield to judicious treatment—first, by simply paying attention to cleanliness, by washing the mouth and teeth, having the cavities filled, foul roots extracted, and the diseases of the gums cured. After these are attended to there yet remain a large multitude of persons who are afflicted, and from whom all hope of cure has fled, as much as if they had entered the Inferno, over whose gates the inscription reads, to leave all hope behind; persons who have no more idea of any cause, of their affliction, by any act of theirs, than they have of the causes of special high tides or the color of the skin of the negro.

We find, in approaching our subject, without going deeper into it than the limits of our time admit, that we must seek for the cause of the odor in emanations of the glands placed along the mucous tract, from its commencement to its close, and, of course, more particularly those of the mouth, throat, and nasal passages. We may also seek for it in the air from the lungs, as an emanation from the blood itself, concentrated and condensed on the air passages by the passing of the air through the respiratory track.

These two sources are sufficient to account for all the trouble, and we need not seek for other causes, even though there may be others; for, if we understand the philosophy of the cause of disease in these parts, we probably shall be able to understand it in all other parts of the system.

In examining the condition of health, we find that the system may, in its relation to food, be divided into three separate functions: First, the mouth, teeth and glands, salivary, tonsilary, etc., which are concerned in the preparation and passage of the food into the stomach, may be termed *organs* of preparation or *reception.* Second, the stomach and a portion of the alimentary canal, with the pancreas, liver and surrounding viscera, may be termed the organs of *assimilation;* and third, the remainder of intestinal canal and the kidneys, with their related organs, may be called the organs of *excretion.*

I maintain that the functions of each organ are strictly marked; that never, under the conditions of absolute health, does one organ interfere with, or accept even, the duties of another; but that, in an overworked or diseased condition of an organ, some other one may assist it in its duties, perform a portion of them, or, perhaps, for a time, the whole of its functions, as for instance, the skin, which, at a venture, we will say should only secrete a lubricating fluid for protecting its surface and adjusting the heat of the body, *may* carry off through its pores the emanations that more properly belong to the kidneys, bladder or intestines. This "interchange of function," well known to physiologists, assists us in understanding our subject; for, if my premises are right, all the impurities of the system should be removed by the *organs* of *excretion,* and will be so removed when they are in a state of health.

What is a state of health? The human system is like a furnace. The food we eat is the fuel. The stomach and the mouth are the cooks and wood-choppers, who prepare the fuel, and the air is the flame that burns it, blown on by the bellows, the lungs. Along the intestinal tract are workmen that throw out the ashes of the burnt fuel into the gut that carries by its slow movement the rejected matter to the verge of the system. Let those workmen be disabled or overworked; let the motions of the rejecting canal be retarded, and the constantly accumulating secretions must find other outlets; an interchange of function takes place and, through the skin and through some of the portions of the mucous tract, the essential poison is poured out; similar organs taking on themselves more easily the interchange of function. The presence of disorganized matter is positively known by the smell. I take this as a fact not to be disputed, that where there is an ever present disagreeable odor there must be disagreeable, or in other words, *poisonous matter* present; matter that should be eliminated, and which is detrimental to health, and, therefore, the organs containing it are in a state of disease.

But here I am met with the objection that some persons are entirely well, with the exception of foul breath, and I must account for that condition separately. Doubting the fact I will, however, try to do so.

Every human being has a certain amount of vitality—some more, some less. *Great vitality signifies great fire, active lungs*, active motions of blood, stomach, heart, bowels and brain. Everything eaten is appropriated, and is burned to ashes; not alone what is best for sustenance, but what is *poor* for sustenance; whatever is put in for fuel. As in the great Boston fire, six years ago, woolen goods burned like oil, and the very slates and the granite itself were burned to ashes. But such vigorous natures are *rare*. Some fires burn slowly. They do not even consume the purest elements of combustion, and cannot burn what is ordinarily non-combustible; and, therefore, such things must be got rid of in some other way if they are placed within the system.

Want of power, then, or weakness in the organs of assimilation, stands first in the way. If the stomach is not able to discharge easily the duty put upon it, irritation of the mucous membrane takes place, which rapidly extends over its surface. What is meant by extending over its surface? It means that the irritation that would be dangerous or fatal to the vitality of the membrane if confined to one spot or, more strictly speaking, to the mucous cells that compose the special surface irritated, by the power of cell contact, or by the intrinsic incorporated power in the cells themselves, distribute the disease or the irritation more widely, thereby reducing its power over any one spot or section of mucous surface.

We have always to remember that irritation produces an *increase of activity.* If a surface is in the condition of secreting any given fluid, slight irritation increases its secreting power. If the irritation is greater, inflammatory action takes place which may reverse the glandular action and, for a time, the secretions may give way to feverish or heated conditions; but we must always remember, and place it as the *corner stone* of our investigations, *that disease is the manifestation of an attempt on the part of nature to cure some ailment.* It is the process of work to rid the system of injury and affliction.

We will take the case of an overloaded stomach for illustration. Persons who are liable to chronic bad breath will have it come on under such a condition. An hour or two after eating a tainted breath is noticed ; it increases rapidly and constantly until the stomach appears to be entirely empty. Even then it may continue and increase, but, if fasting is continued long enough, it disappears and purity returns.

I have said that the organs below the stomach should perform all the acts of secretion. If they perform their nominal functions thoroughly, there will not be any taint of breath; for the food, after remaining in the stomach a certain length of time, will be passed from it into the small intestines, where all unfinished processes will go on to their completion, or it will rapidly be passed to the more especially excretory organs; but with the greater portion of persons, who live on the average diet of Americans who reside in our cities, with their sedentary habits and want of vigorous labor or play, this is not the case. The fire which should burn brightly, that should burn all fuel to clean ashes, smoulders and smokes, and the mucous membrane, which cannot absorb the weighty *debris* of the system, nevertheless endeavors to assist in carrying off the superfluous load. It does this by a vicarious exchange of function, taking a share of the work, by carrying off some of the smoke and foul gases produced by the imperfect combustion, or, in other words, imperfect digestion, and the fermentation of more food than the stomach can wrestle with.

The proper balance of diet in its vegetable, animal, and mineral constituents will, as far as diet is concerned, produce health; but it is necessary that all the surplus that is not retainable as nourishment and fat be thoroughly eliminated from the system; but to do this the excretory organs must be in *active working condition.* It must not be necessary to coax them to perform their duties, but they should be in condition to command obedience with a force that must be at once obeyed.

If these organs are not capable of this duty, and cannot fulfil it completely, some portion of the *debris* must remain in the system; and as it is poison, as it contains in itself the elements of death, it cannot be stored in any one organ without danger of fatal sickness.

Nature by the same mode of interchange of function distributes it throughout all the organs that possess any excretory powers. She loads the blood with it; she lines the air-passages of the lungs with it; she forces it out through the skin. Wherever she can oxydize it she tries to do it; for that destroys its action. But where it accumulates in large amounts, it is liable to a sort of spontaneous combustion, attacking veritable tissue—and especially the delicate tissues of the lungs—burning and destroying them like a fire. Things at first unattackable, get involved; heat produces heat; the firm tissues that resist ordinary attacks burn, and the currents of the blood like untoward winds scatter the firebrands through the whole organization. This is consumption; but mark this important fact, for it is probable that you hear it announced for the first time, that pulmonary *consumption is a secondary disease, the primary cause of which is defective excretion*, and it has its primary seat in the excretory organs. When physicians adopt this view of mine and direct their observations to this cause, they will find that there is a *cure* for this dreadful disease.

I said Nature loads our blood with *debris*. How do I know it? By the fact that it can be smelled in the blood. The normal blood has an odor but not the rank, disagreeable smell we sometimes find coming from the blood of some wounded persons. To the mucous membrane she seems to have delegated, when necessary, the province of carrying off first bad odors and secondarily mucous deposits.

I have spoken of this as an interchange of function; but when we consider that the mucous membrane lines the whole intestinal track, the whole internal, preparative, assimilative and excretive track, as far as the alimentary canal is concerned, and that the mucous membrane of the bowels is so intimately connected with the excretion of *highly offensive odors*, the office may be said to be one of the normal duties of that organ or membrane.

Whether my theories are correct or not, it is a fact that certain habits will bring on and continue foul breath, and I am convinced that the largest cause is found in constricted, irregular and costive habits of bowels, and that for its cure particular attention to regulating this portion of the system is absolutely necessary.

The next cause, and intimately connected with the other, is overloaded and dyspeptic conditions of the stomach. This fact is easily demonstrated. Let any one who has the tendency to inflamed condition of the mucous coat of the mouth, throat and surrounding parts, eat an over-full meal of even the simplest substances, under conditions that do not favor the digestion of it, and it will bring on the trouble. A hearty meal just before bed-time, will probably produce it during the night, so that on awakening in the morning the mouth will be noticeably dry and foul. So also will the tendency be kept up by *too frequent meals.* It were better that we all had an interval of six hours between each meal. The stomach should have time to rest, and food must have time to digest; articles taking *five* hours time *cannot* be digested in *four* hours, and no new food should be mixed with the partly digested material. This, I am sure, is one of the *most fruitful causes* of inflamed mucous membrane and bad breath.

But if the presence of food at the wrong seasons and in over quantities produces trouble, how much more so will wrong food in great quantities stimulate and produce it? I cannot do better than to refer any one interested, to the well known tables of digestion founded on Dr. Beaumont's experiments with Alexis St. Martin, for the only positive knowledge we have of digestion in the live, human stomach, and also to his "experiments," crude and unmethodical as I think they often were, as proving some important facts regarding inflammation produced by various substances, foods, condiments, &c. A large essay could be written on this single point of our subject, but I can only glance at it.

*Spices and heating condiments*, as a rule, should be avoided. Under certain circumstances they may be used as medicine, but as food, and as pleasurable excitants by one who has the tendency named, they should be shunned. Let me give the reason *why*; for I am sure I have never heard it properly given. Spices form compounds with animal and vegetable substances, thereby retarding or preventing decay; or in other words they prevent the rapid separation of food into soluble parts, and being able to do it outside of the system, will, in the same manner, prevent rapid digestion in the food receptacles, which is a process not entirely unlike decomposition—the first process of decay. Let me advance a step further and give a *rule of digestion* discovered by myself. Rule: All articles of food easily decomposed by warmth and moisture out of the human stomach are easily digested in the stomach; and all articles which are slow to decompose under such circumstances, are slow of digestion; the ratio of digestive process being proportionate to the process of decomposition.

This rule holds good with some few exceptions.

*Large quantities of fluids* are irritants to some persons, by *floating food* and preventing digestion. I am certain that fluids are often difficult of absorption, and remain in the stomach a long while, probably from some inaction of the secretory glands. I refer to the simple fluids, such as *water*, *milk*, *soup*, *etc.*, but the presence of stimulating drinks, as rum, brandy, gin, etc., have a well known effect, and will produce, almost immediately in some cases, by irritating the mucous membrane, a bad taste in the mouth and bad breath.

I have spoken of the bad odor of the breath being a sort of *smoke of unburnt food*, but the continuance of this disease is, I am sure, always accompanied by phlegm and catarrhal troubles. When the membrane cannot discharge from itself the load that is placed upon it, it clogs up and the secretion becomes viscid and abundant. It attaches itself to the surface in a ropy, adhesive mass. It is a carbonaceous deposit, and would indicate, it seems to me, the presence of too much carbonaceous matter in our food, that the system is trying to get rid of; and I suggest that the diet should be deprived of a portion of such matters, in the form of grease, butter and milk, and that in their places be substituted fruits, or peas, beans, and green vegetables, or what is better still, if one can get excercise or vigor to digest them, turnips, radishes, celery, and all the anti-scorbutics.

I must here mention one other cause of trouble; the presence of unmasticated food, half-chewed, bolted, gobbled down, any way it can be got into the stomach. Observation leads me to think that nine-tenths of the trouble arising from the use of various articles of food *denominated unhealthy*, is from want of *proper mastication;* and I would also lay down this rule to parents. Rule: If you want your children healthy, teach them to chew their food fine and eat slowly. Do you think they can learn it themselves? So can they learn to read and write, to comb their head and brush their teeth; but how much better can they do these things with instruction? And why should the important instruction in chewing food be neglected? Is it not more important than music or dancing, or the infantile theology that is so much taught, or the molecular theory taught to my twelve-year old girl?

Everything that *disturbs the digestion*, either from *internal* or EXTERNAL *causes* will *prevent* the cure of this disease. *Want of exercise*, *too much worry and mental strain*, too much sensuality, all conspire to prevent the physiological fires from consuming the fuel without smoke.

To those to whom this disease comes as an affliction, we must tender our regrets; they can study the hints here thrown out: they can know they have our sympathies; but from those who nasty their mouths by their own filth—by uncleanliness, by vile and dirty habits, we must withhold them. We are members of a profession that teaches cleanly habits, and to make our teachings effectual we must be cleanly ourselves. Lenient as I am, and as I hope all of us may be to such, I cannot conceive how any dentist can approach his patients with a befoulment of fresh garlic, onions or tobacco in his mouth; but disagreeable as this is or may be to most persons, it is yet weak and unobnoxious compared with the nastiness of garments and mouth filled with the stale, second-hand odors of strong cigars or pipes that some of my friends are in the habit of using, and I am sorry to say that they are as innocent of their faults in this respect as babies. Let me draw you a picture.

Some years ago I knew a young dentist who smoked tobacco. He did it genteely. He smoked a very mildflavored cigar. He drove into the city, and when he arrived at his office, he put away his garments carefully, and he thought he disguised the habit from his patients; they did not complain. He did not desire to surrender the habit, and he did not wish to offend his patients. *He thinks he does just the same thing now!* I doubt that any one here could convince him to the contrary, and he will think that I am joking, and do not, cannot mean him, so slowly has the vile habit crept on him. To-day he smokes a stinking thing; rank, outrageous, which is an offence to good taste, decency and manners. He should mount to his attic and there fume away if he must; but no, he will stand and blow his villainous smoke square in your face. He forgets good taste and walks the street with a cigar in his mouth; and when he enters the presence of ladies he carries with him a feeling of disgust and a nasty smelling breath, that is as hard for *them* to bear as some of the breaths of his *highest flavored patients* are for *him*. What excuse is there for such a man who outrages decency in such a manner, and talks to his patients loudly about brushing their teeth and keeping their mouth clean? There is just one man worse than this one. It is he who adds this befoulment to an already stinking breath.

But I must sum up my remarks and then close, first noticing the fact that there is an important item yet to be investigated; and that is whether foul breath is a disease which is catching, or can be conveyed from one to another. I am of the opinion that it is. In a system without any tendency that way, the over-laden breath may pass into the lungs from another person and no bad results be noticed; but as is probably the case in conditions favoring diseases of this sort, there are spores or seeds of disease, or there are *vibriones*—minute animal life floating in air and feeding on, as well as producing diseased conditions that may pass from mouth to mouth and breed the same disease by contact in another system. I have had one or two attacks of disordered mucous membrane after working for persons of peculiarly foul breath; and although I cannot say positively that I can trace it to that cause, I am and have been seriously suspicious that it had much to do with it.

I have not in this essay proposed any course of medical treatment; and yet in some cases I have no doubt that a mild purgative or cathartic would assist temporarily in removing the trouble; but my theory of medicine is, that if the articles we call medicines, which consist to a great extent of concentrated bitter herbs, could have been supplied with our food, i. e., if they had not been purged from our food, or the portion of food producing the same effect in the natural way been taken from our diet, we should go along pretty well without purgatives of any sort.

But what palliative shall the dentist have who, when bending over his patient suddenly discovers that there is an unpleasant taint on his breath? The first thing he must do, and as a rule, *do it always*, when working for a patient, is to *close his mouth tight and breathe through his nose.* Have at hand some disinfectant, as lime-water and chalk, chlorate of potash in solution, or in the dry powder, Burnett's milk of magnesia or some other good mouth wash, and every chance he can get, make for the remedies, rinse the mouth rapidly and silently and run back to his patient, and keep his mouth closed. Another remedy or palliative is to keep the mouth wet with fresh saliva. This can be done by chewing some inodorous thing, a piece of paper, wood, or better still, a small piece of gum elastic; the flood of new saliva will temporarily drown out the excretions. Do not use snake-root, cardamon seeds and similar drugs, for the remedy is often as bad as the disease; rinse with cool water, and try the bit of elastic gum, taking it out when your patient has gone.

Cure the disease if you can! This is important. *Increase* your *vigor* by *exercise*, and *abstinence* as well. PLAY!! I wish we Americans could *play*. Play ball as a *frolic*, and not as hard work; not at the risk of broken fingers and bunged eyes, but for the *fun* of the thing, and *dance.* Inhale and exhale the pure air; oxydize the food and blood; *that* will kill the odors.

What result but a *fatal* one must come from the inhalation of our own diseased *emanations*? Hour by hour, day by day, and night by night drawing in with each breath such horrid odors, what but consumption will result from it?

I know not if any statistics can be found on the subject, but I will venture to say that continuous foul breath must finally increase the consumptive tendencies of those who have it, and for two reasons. First, it shows a weak tendency in the individual, and, secondly, it will react on itself by returning the odors with their poison into the system.

Let me finish here, by asking the members of our profession to give more attention to this important subject, by thanking you for your attention, and by urging you not to accept superficial answers to inquiries on all physiological questions, but to think for yourselves and not be satisfied with the mere outward descriptions of organs, but to go further and construct a *philosophy of physiology*, as I have tried to do, which shall be as a map for you by which you shall see the way of the workings of nature in the human system, and make normal and diseased action plain and simple.

It will give me pleasure, some time, to explain to you how far I have satisfied myself on this subject, and what points are in doubt, in my mind; but I have satisfied myself that the *philosophy* of physiology is extremely simple.—*Missouri Dental Journal.*

